# Improved cytocompatibility and antibacterial properties of zinc-substituted brushite bone cement based on β-tricalcium phosphate

**DOI:** 10.1007/s10856-021-06575-x

**Published:** 2021-08-18

**Authors:** Inna V. Fadeeva, Margarita A. Goldberg, Ilya I. Preobrazhensky, Georgy V. Mamin, Galina A. Davidova, Nadezhda V. Agafonova, Marco Fosca, Fabrizio Russo, Sergey M. Barinov, Simona Cavalu, Julietta V. Rau

**Affiliations:** 1 A.A. Baikov Institute of Metallurgy and Materials Science, Russian Academy of Sciences, Leninsky pr. 49, Moscow, Russian Federation 119334; 2 Department of Materials Science, M.V. Lomonosov Moscow State University, Leninskie Gory 1, Moscow, Russian Federation 119991; 3 Kazan Federal University, Kremlevskaya 18, Kazan, Russian Federation 420008; 4 Institute of Theoretical and Experimental Biophysics of Russian Academy of Sciences, Institutskaya 3, Pushchino, Moscow, Russian Federation 142290; 5 G.K. Skryabin Institute of Biochemistry and Physiology of Microorganisms, Russian Academy of Sciences, Federal Research Center “Pushchino Scientific Center for Biological Research of the Russian Academy of Sciences”, pr. Nauki, 5, Pushchino, Moscow Region, Russian Federation 142290; 6 Istituto di Struttura della Materia, Consiglio Nazionale delle Ricerche (ISM-CNR), Via del Fosso del Cavaliere 100, 00133 Rome, Italy; 7 Department of Orthopaedic and Trauma Surgery, Campus Bio-Medico University, Via Alvaro del Portillo 200, 00128 Rome, Italy; 8 Faculty of Medicine and Pharmacy, University of Oradea, P-ta 1 Decembrie 10, 410073 Oradea, Romania; 9 Department of Analytical, Physical and Colloid Chemistry, Institute of Pharmacy, I.M. Sechenov First Moscow State Medical University, Trubetskaya 8, build. 2, Moscow, Russian Federation 119991

## Abstract

For bone replacement materials, osteoconductive, osteoinductive, and osteogenic properties are desired. The bacterial resistance and the need for new antibacterial strategies stand among the most challenging tasks of the modern medicine. In this work, brushite cements based on powders of Zinc (Zn) (1.4 wt%) substituted tricalcium phosphate (β-TCP) and non-substituted β-TCP were prepared and investigated. Their initial and final phase composition, time of setting, morphology, pH evolution, and compressive strength are reported. After soaking for 60 days in physiological solution, the cements transformed into a mixture of brushite and hydroxyapatite. Antibacterial activity of the cements against *Enterococcus faecium, Escherichia coli*, and *Pseudomonas aeruginosa* bacteria strains was attested. The absence of cytotoxicity of cements was proved for murine fibroblast NCTC L929 cells. Moreover, the cell viability on the β-TCP cement containing Zn^2+^ ions was 10% higher compared to the β-TCP cement without zinc. The developed cements are perspective for applications in orthopedics and traumatology.

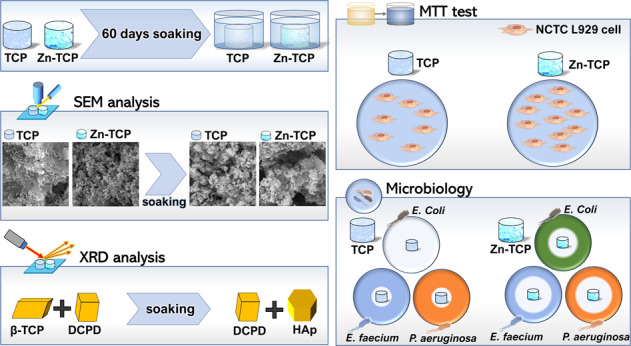

## Introduction

Currently, the need to manufacture bone implants is highly requested for several bone defects in the human body [1]. Indeed, the incidence of bone deficiencies associated with fractures, surgical resections, and osteoporosis is rapidly growing [2]. Commonly, substitutional materials for bone tissues rely on the use of autologous grafts, which represent the “gold standard” of bone substitution [3]. However, their use is limited by donor site morbidity, available quantities (especially when bone defect is large), higher risk of infection, and other complications [4]. Allogeneic bone is related to limited donor source and safety problems [5]. In order to overcome these limitations, synthetic bone materials have been developed as valid alternative to autologous grafts being able to reduce infections and complications. Moreover, there is an increasing need of clinically available and degradable implants in orthopedics [6].

Indeed, implants can be divided into two large groups: non-biodegradable metals and alloys (for example, titanium, cobalt and their alloys, steel, etc.), and biodegradable materials. Non-biodegradable metal implants have been used for more than 100 years for healing bone defects and trauma, but they need to be removed after healing and/or often be replaced upon healing by an additional surgery [7]. The most promising bone implant materials are biodegradable ones, more suitable in some clinical applications such as fracture treatment, resorbed in the human body and can be substituted with time by the natural bone tissue, avoiding repeated surgical interventions. An ideal bone replacement material should be osteoinductive, osteoconductive, and osteogenic. The main concern for the use of synthetic materials for bone reconstruction is their ability to integrate and vascularize in the body.

The most common types of calcium phosphates applied for synthetic bone implant materials are hydroxyapatite (HA), tricalcium phosphate (TCP), and biphasic calcium phosphates. The advantage of TCP is that it is a rapidly resorbing ceramic with respect to HA that resorbs slowly. TCP can be used for the treatment of bone defects, dental materials, biomedical cements, implant coatings, and other applications [8–13].

Depending on the temperature, TCP exists in the form of three polymorphic modifications: α, β, and β′. Only two modifications are stable at room temperature: α-TCP and β-TCP. α-TCP crystallizes in a monoclinic crystal lattice, while β-TCP in a rhombohedral one. Both polymorphic modifications are used in medicine, but due to the difference in physical and chemical properties, their application areas are different. In aqueous media, α-TCP hydrolysis leads to the formation of calcium-deficient HA. Due to the high reactivity of α-TCP, it is often used as the main powder component in bone cements. β-TCP is widely used to manufacture mono—or biphasic ceramics and can also be applied for bone cements [14–19].

Relevant clinical issues for biomedical implants concern osteointegration, inflammation, and infections. Biomaterial associated infection is a devastating complication in orthopedic and trauma surgery that often leads to multiple surgeries, pain, functional losses, increased morbidity, and even mortality. Thus, in this scenario, the development of new biomaterials with improved antimicrobial properties that prevent bacterial adhesion, colonization, and proliferation is promising.

Zinc (Zn) is an important biological trace element that plays a role in the normal growth and development of the skeleton. Its content in human bones (0.0126–0.0217 wt%) is about 28% of the total amount of Zn in the body (0.0030 wt% of Zn in tissues) [20]. The lack of Zn slows down the growth of the bone mass and has a negative influence on the bone metabolism [21]. On the other hand, Zn deficiency is a factor of risk for bone osteoporosis [22]. Also, it is an essential trace element for promotion of osteoblast differentiation and proliferation [23], and it is a component of many metallo-enzymes and proteins including alkaline phosphatase [24]. An ideal bone replacement material should stimulate the growth of natural bone tissue. For this purpose, it is possible to use growth factors as additives to bone cements. However, growth factors have high cost [25] and undesirable side effects [26, 27]. The addition of ions is often considered as an alternative way with respect to the use of growth factors. They are not only significantly cheaper, but also reduce the probability of negative side effects on the body. Thus, taking into account an important role of Zn in the human body described above, the introduction of Zn^2+^ ions should have additional positive effects, stimulating the formation of native bone. Furthermore, Zn is well-known to possess antimicrobial properties [28, 29]. Therefore, introducing Zn in biomaterials could provide antimicrobial properties [30], avoiding infections during surgery.

Previous research showed the possibility of replacing calcium in calcium phosphates with Zn ions. In [31], the preparation and physical-chemical characterization of Zn-substituted calcium phosphate powders was performed. Authors [31] demonstrated that up to about 10 at.% (about 15 wt%) of Zn ions do not significantly influence the crystal lattice of HA and brushite, whereas authors [32] showed that Zn can replace calcium in the β-TCP structure up to about 10 at.% with the preference of the Ca(5) site.

Substituted brushite cements are good candidates for use in restoration of bone and periodontal defects, and some issues regarding their clinical applications and trends are reviewed by authors [33]. Authors [16] reported Zn-substituted brushite cements and investigated their antibacterial activity. It was demonstrated that 0.6 wt% of Zn substitution leads to the inhibitory effect toward *Escherichia coli*. It should be stressed that the suitable amount of substitution ion is an important issue. The cytotoxicity with respect to osteoblastic and other cells depends on the concentration of Zn. Authors [34] report that for β-Zn-TCP ceramics, the Zn content higher than 1.20 wt% causes cytotoxicity to osteoblastic (MC3T3-E1) cells. For α-Zn-TCP powder, with concentration of Zn lower than 0.11 wt%, the cytocompatibility was approximately the same as that for the pure α-TCP powder [35]. According to [36], the optimum Zn content in α-Zn-TCP is around 0.03 wt%, and this material was able to stimulate more bone formation with respect to the pure α-TCP.

The present study aimed to develop Zn-substituted brushite cement with a simplified recipe and improved characteristics. A resorbable brushite bone cement with neutral acidity, suitable for surgical on-site use, is reported. Instead of β-TCP (as in the previously obtained brushite cement based on Zn-substituted TCP [16]), as initial powder we used an equimolecular mixture of β-TCP and monocalcium phosphate monohydrate (MCPM), and as hardening liquid (instead of a liquid based on single-substituted magnesium phosphate with orthophosphoric acid [16]), we used 8% of aqueous solution of citric acid containing 30% of ammonium citrate. Here, as a suitable substitution ion amount we propose a brushite cement based on Zn-substituted β-TCP with a concentration of Zn of 1.40 wt% and investigate its physical, chemical, and biological properties, including the cytotoxicity study with the NCTC L929 fibroblast cells from murine subcutaneous connective tissue. Introducing a suitable concentration of Zn^2+^ substitution we aim to impart the developed cements also with antibacterial properties and assess them against bacteria strains of *Enterococcus faecium, Escherichia coli*, and *Pseudomonas aeruginosa*. The antibacterial characteristics of the cement are of particular importance for orthopedic applications in view of bacterial resistance issue and the need for new antibacterial strategies, standing among the most challenging tasks of the modern medicine.

## Materials and methods

Zn-substituted and non-substituted TCP powders were obtained by heterogeneous phase synthesis using mechanochemical activation [14]. A planetary mill with the rotation speed of 15,000 rpm was used as an activator. For synthesis, powders of freshly calcined calcium oxide (chemical grade, Chimmed, Moscow, Russia), ammonium dihydrophosphate (chemical grade, Chimmed, Moscow, Russia), and zinc nitrate (chemical grade, Chimmed, Moscow, Russia) were placed in Teflon container, taken in molar ratios, calculated from the reaction Eq. (1). The interaction of components was carried out according to the following schemes (1, 2):

1 $$\begin{array}{l}2,9{{{\rm{Ca}}}}\left( {{{\rm{{NO}}}}_3} \right)_2\, + \,0,1{{{\rm{Zn}}}}\left( {{{\rm{{NO}}}}_3} \right)_2\, + \,2\left( {{{\rm{{NH}}}}_4} \right)_2{{{\rm{HPO}}}}_4\, + \\ 2{{{\rm{NH}}}}_3 \cdot {{{\rm{H}}}}_2{{{\rm{O}}}} \to {{{\rm{Ca}}}}_{2.9}{{{\rm{Zn}}}}_{0.1}\left( {{{\rm{{PO}}}}_4} \right)_2\, + \,{{{\rm{6NH}}}}_4{{{\rm{NO}}}}_3 + 2{{{\rm{H}}}}_2{{{\rm{O}}}}\end{array}$$

2 $$3{{{\rm{CaO}}}} + 2\left( {{{\rm{{NH}}}}_4} \right)_2{{{\rm{HPO}}}}_4 \to {{{\rm{Ca}}}}_3\left( {{{\rm{{PO}}}}_4} \right)_2\, + \,4{{{\rm{NH}}}}_3 + 3{{{\rm{H}}}}_2{{{\rm{O}}}}$$

Corundum balls for material milling in a planetary mill (MP 4/1, “Technocentr” Ltd, Rybinsk, Russia) were taken in the ratio of 1:5. After 20 min of mixing, 300 ml of distilled water was added to the mixture, and the milling was continued for other 20 min. After that the suspension was separated from the balls, and the resulting product was filtered out and dried at 105 °C. To obtain cement powder, the Zn-substituted TCP powder was pre-calcined at 900–1100 °C, disaggregated by passing through a sieve with a cell diameter of 400 µm, and mixed with MCPM (Sigma-Aldrich, St. Louis (Missouri) USA) in a molar ratio of 1:1 [37]. The hardening liquid was prepared by dissolving 30 g of ammonium citrate and 8 g of citric acid in 62 ml of water. Cement samples were obtained by mixing powder of Zn-substituted or non-substituted β-TCP and MCPM with the hardening liquid in a ratio of 3:1. A teflon cylindrical mold with a diameter of 8 mm was used for forming the samples.

Samples were hardened in 100% humidity during 24 h. The phase composition was investigated by the X-Ray Diffraction (XRD) method. Phase analysis of the obtained compounds was performed on a Shimadzu XRD6000 diffractometer (Kyoto, Japan) (CuKαλ = 1.5405 Å radiation, JCPGS file data).

The Zn content in the powders and in the cements was determined using an emission optical spectrometer with inductively coupled plasma Optima 5300 DV.

The setting time for cements with the ratio of cement powder: hardening liquid = 3:1 was 8 min, which is optimal for the preparation of cement paste during surgical operation.

The hardening time was determined using the Vicat device “OGC-2” for determining the setting time of cements (Ekaterinburg, Russia) by immersing a needle with a cross-section area of 1 mm^2^ with a load of 100 g in the cement paste formed after mixing the cement powder with the hardening liquid. The cement was considered solidified when the needle did not leave a sign on the sample.

For the pH measurement, a cement sample of 0.5 g was immersed in 1 ml of distilled water. The pH of the cement was measured using a glass combined electrode, which was lowered into the solution formed over the cement sample. Measurements were carried out by the pH-meter Expert-001 (Moscow, Russia). Brushite cement based on β-TCP and Zn-containing brushite cement based on Zn-substituted β-TCP were examined during the experiment.

The cements’ solubility was studied for samples based on β-TCP and Zn-β-TCP, soaking them in 0.9% sodium chloride solution containing TRIS buffer (Chimmed, Russia) at 37 °C for 60 days.

The microstructure of cement samples was studied by a scanning electron microscope (SEM) (Tescan Vega II, Brno, Czech Republic) with energy dispersion X-ray INCA spectrometer. The accelerating voltage of the electron gun was 17–21 kV. Cement samples based on Zn-substituted and non-substituted β-TCP were placed in physiological solution for 60 days, after which changes in the microstructure were investigated.

Fourier transform infrared spectroscopy (FTIR) spectra of the synthesized compounds were obtained for samples in mixtures with potassium bromide using the Nikolet Avatar-330 spectrometer (Nikolet Instrument, Madison, USA) in the range of 400–4000 cm^−1^.

For mechanical testing, five cylinders with a diameter of 8 mm (+/0.1 mm) and a height of 16 mm (+/0.1 mm) were prepared for each cement type: β-TCP and Zn-substituted β-TCP cement. The mechanical strength of cement samples under compression was measured 7 days after cements setting using the mechanical machine Instron 5800 (Norwood (MA), USA).

Conventional (continuous wave) electron paramagnetic resonance (EPR) were carried out by a Bruker Elexsys 580/680 spectrometer in the X-band (ν_MW_ = 9–10 GHz). In order to generate paramagnetic centers in the original material, X-Ray irradiation of the initial powders was made by the URS-55 source (*U* = 55 kV, *I* = 16 mA, W anticathode) with the estimated dose of 20 kGy at room temperature for 60 min.

In accordance with the requirements of ISO 10993.5-2011 [38, 39], the study of cytotoxicity of materials was carried out using extracts of materials in Dulbecco’s modified Eagle’s culture medium (DMEM) (Sigma-Aldrich, St. Louis (Missouri), USA) with the addition of 100 units/ml of penicillin/streptomycin (Sigma-Aldrich, St. Louis (Missouri), USA). All the samples were subjected to sterilization at 180 °C before the test. The extracts preparation was performed under aseptic conditions at 37 °C for 3 days. For each material, three samples were used. The ration of the material surface area to the DMEM volume was 3 cm^2^/1 ml. The NCTC L929 fibroblast cells of murine subcutaneous tissue were provided by the Institute of Cytology of the Russian Academy of Sciences (Moscow). The NCTC L929 cells were seeded into the wells of 96-well plate at a concentration of 30,000/cm^2^ in DMEM, containing 5% of fetal bovine serum (Sigma-Aldrich, USA). After 18 h, the medium was replaced by 100 µl of extracts of the tested materials and cultured at 37 °C in the atmosphere of 5% CO_2_ for 24 h. After that, extracts of materials were investigated for cytotoxicity using the MTT test consisting in the reduction of tetrazolium salt (3-[4,5-dimethylthiazole-2-yl]-2,5-diphenyltetrazolium bromide, MTT) by mitochondrial and cytoplasmic dehydrogenases of active alive cells and resulting in the formation of blue formazan crystals, which are soluble in dimethylsulfoxide.

Antibacterial activity was assessed for the *E. coli*, *E. faecalis*, and *P. aeruginosa* bacteria species. The bacteria were grown in the Mueller Hinton Agar (HiMedia, India) nutrient medium. First, cement samples were sterilized by UV radiation for 30 min. Antibacterial activity was tested by positioning the materials’ disks on a freshly seeded bacteria lawn. The bacteria concentration of 10^6^ colony forming units/ml (100 µl per dish) was used. Petri dishes with bacteria and disk samples were situated in a thermostat and cultured for 18–24 h. The antibacterial activity was determined from the width of bacteria zone of inhibition.

Statistical treatment of the results was performed using the Origin program “Origin Pro 2016 (64 bit)” (OriginLab Corporation, Northampton, MA, USA), the error was taken as a standard deviation from the average value, and the differences were considered reliable, according to the Mann–Whitney *U* criterion, at *p* < 0.01.

## Results and discussion

According to the elemental analysis, the Zn amount in the β-TCP powder was 6.40 wt%, while in the cement—1.40 wt%.

**Fig. 1:**
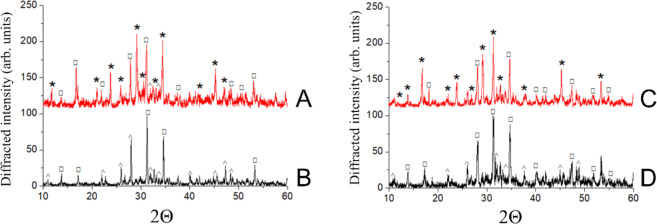
Diffractograms of: TCP-based cement (**A**) before and (**B**) after soaking in physiological solution and Zn-substituted TCP cement (**C**) before and (**D**) after soaking in physiological solution. Labeled peaks relate to0 (*) β-Ca_3_(PO_4_)_2_ (TCP) (card [09-169]), (□) CaHPO_4_·2H_2_O (DCPD) (card [72–713]), and (^) Ca_10_(PO_4_)_6_(OH)_2_ (HA) (card [72–1243])

The pH values of the β-TCP and Zn-β-TCP cements were determined at different time points starting from their setting, from 1 min up to 60 min. It was found that immediately after setting the cements were characterized by a weak acidity, i.e., pH = 5.5. After 60 min, the pH increased to 6.5 due to the continued interaction between the cement powder and hardening liquid, since the dissolved ammonium citrate interacted with the calcium-containing components (β-TCP and MCPM) to form non-soluble calcium citrate on the surface of these components. Calcium citrate nanoparticles covered the surface of β-TCP and MCPM, slowing down the chemical interaction between them. The increase in pH was due to the fact that the amount of the acidic component of MCPM decreased during the chemical interaction with TCP and, therefore, the concentration of hydrogen ions decreased as well. According to the XRD results shown in Fig. 1A, C, the final phase of cements after hardening was composed of dicalcium phosphate dihydrate, or brushite (DCPD, CaHPO_4_·2H_2_O) and β-Ca_3_(PO_4_)_2_. No impurity phases corresponding to zinc salts were found. The interaction between the components of the cement powder occurred according to Eq. (3):

3 $${{{\rm{Ca}}}}_3\left( {{{\rm{{PO}}}}_4} \right)_2\, + \,{{{\rm{Ca}}}}\left( {{{\rm{{H}}}}_2{{{\rm{PO}}}}_4} \right)_2\, + \,8{{{\rm{H}}}}_2{{{\rm{O}}}} \to 4{{{\rm{CaHPO}}}}_4 \cdot 2{{{\rm{H}}}}_2{{{\rm{O}}}}$$

After soaking in physiological solution, the HA phase was detected in the XRD patterns, along with DCPD (see Fig. 1B, D). This is associated with the transformation of DCPD into HA, according to the following scheme (4):

4 $$10{{{\rm{CaHPO}}}}_4 \cdot 2{{{\rm{H}}}}_2{{{\rm{O}}}} \to {{{\rm{Ca}}}}_{10}\left( {{{\rm{{PO}}}}_4} \right)_6\left( {{{\rm{{OH}}}}} \right)_2\, + \,4{{{\rm{H}}}}_3{{{\rm{PO}}}}_4 + 18{{{\rm{H}}}}_2{{{\rm{O}}}}$$

A semi-quantitative comparison between the peak positions of the experimental diffraction patterns showed that Zn substitution in the TCP lattice of powders did not induce any particular position shift (see Table 1). This is probably due to the relatively small amount of the introduced Zn (1.4 wt%) that led to a negligible modification of the TCP lattice parameters. This is in agreement with a previous study [32], where a decrease of only 0.7% of lattice parameters was detected with the introduction of 5 at.% (about 8 wt%) of Zn into the TCP lattice.

**Table 1:** Peak position and the corresponding shift of experimental diffraction patterns of β -TCP and Zn- β-TCP powders

2Θ TCP	2Θ Zn-TCP	Δ2Θ
11.72	11.14	−0.58
23.8	23.84	+0.04
27.86	28.06	+0.20
32.46	32.36	−0.10
45.24	45.24	0

**Fig. 2:**
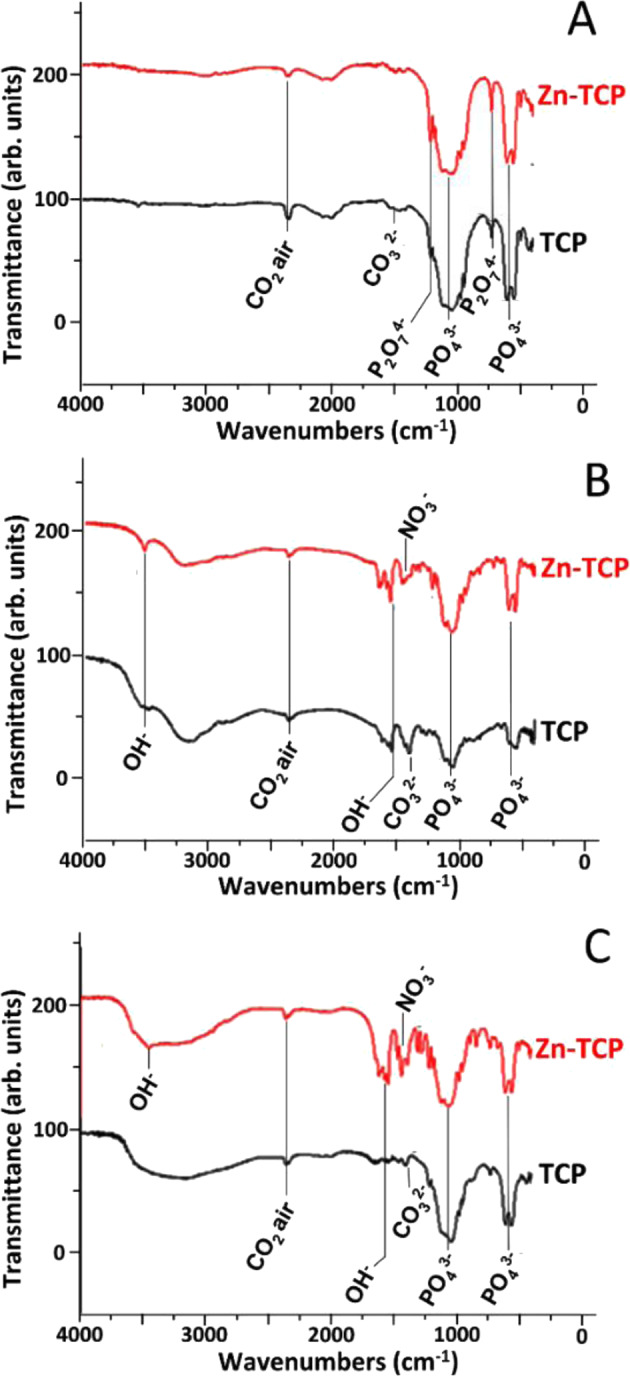
FTIR spectra of: **A** non-substituted and Zn-substituted TCP powders, **B** non-substituted and Zn-substituted TCP cements, and **C** non-substituted and Zn-substituted TCP cements after soaking in physiological solution

The FTIR spectra were obtained for Zn-substituted and non-substituted powders, cements, and cements after soaking in the physiological solution for 60 days, all shown in Fig. 2. In Fig. 2A, the FTIR spectra of Zn-substituted and not substituted powders seem to be very similar.

The regions of the most intense vibrations corresponding to the PO_4_^3−^ group (ν_4_) are highlighted at 565, 603, and 900–1100 cm^−1^ [40, 41]. In addition, a small band at 700 cm^−1^ and a small shoulder of the broad band at 900–1100 cm^−1^, both attributed to the P_2_O_7_^4−^ group from pyrophosphate, were detected, likely due to some thermal decomposition of brushite according to the reaction (5):

5 $$2{{{\rm{CaHPO}}}}_4 \cdot 2{{{\rm{H}}}}_2{{{\rm{O}}}} \to {{{\rm{Ca}}}}_2{{{\rm{P}}}}_2{{{\rm{O}}}}_7 + 5{{{\rm{H}}}}_2{{{\rm{O}}}} \uparrow$$

In the FTIR spectra of cements shown in Fig. 2B, the peaks corresponding to deformation oscillations in the hydroxyl group can be distinguished at 3570 and 632 cm^−1^ [41, 42]. In addition, the peaks at 1300–1550 cm^−1^ related to the CO_3_^2–^ group [42] are well visible. The spectra of cements after soaking, presented in Fig. 2C, are characterized by more intense bands related to the PO_4_^3−^ groups. In all the spectra, the band at 2355 cm^−1^ is present, it can be attributed to CO_2_ from the air. Its appearance is due to some features of the samples preparation procedure [43].

TCP powders were investigated by EPR. Nominally “pure” TCP as well as Zn-substituted TCP, according to their chemical formulas, are EPR silent. In the investigated samples, a small amount (*x* < 0.001) of manganese (Mn^2+^) ions was revealed by EPR as six lines, due to the hypefine interaction between the Mn electron spin and ^55^Mn nuclei (*I* = 5/2) with *A* = 9–10 mT [28, 44, 45]. No other EPR signals were observed in the investigated samples that is a sign of the absence of additional paramagnetic impurities.

**Fig. 3:**
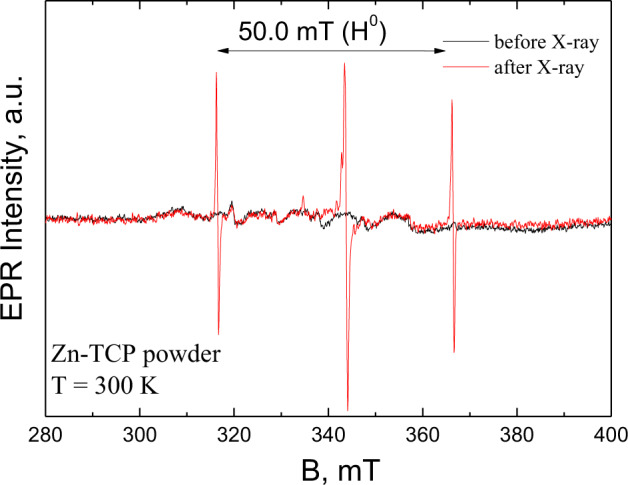
Comparison of EPR spectra for Zn-TCP powder sample annealed at 900 °C before and after X-ray irradiation

The signal appeared after the X-ray influence (Fig. 3). Its intensity in sample annealed at 900 °C was two orders of magnitude lower than that in the TCP annealed at 400 °C that indicates the efficiency of annealing to remove impurities. Further, we will discuss in detail the data obtained only for the Zn-substituted TCP powder sample annealed at 900 °C.

Figure 3 shows that two groups of the EPR signals were detected. One (a pair of lines separated by 50.0 mT) is very often obtained for HA and TCP synthesized by solid state reaction [44, 46] and belongs to the hyperfine structure for the trapped hydrogen—H^0^ stable radical with *I* = 1/2 [47, 48]. In contrast with the results of [46], the hyperfine constant A was found to be higher (50.0 vs. 49.9 mT) and no additional splittings were observed in our experiments. In [47], it was supposed that H atom is trapped in β-TCP at the interstitial site between the two groups of PO_4_^3−^ in the B column. Our data allow suggesting that in the investigated Zn-TCP sample, H is trapped rather at substitutional sites of TCP.

**Fig. 4:**
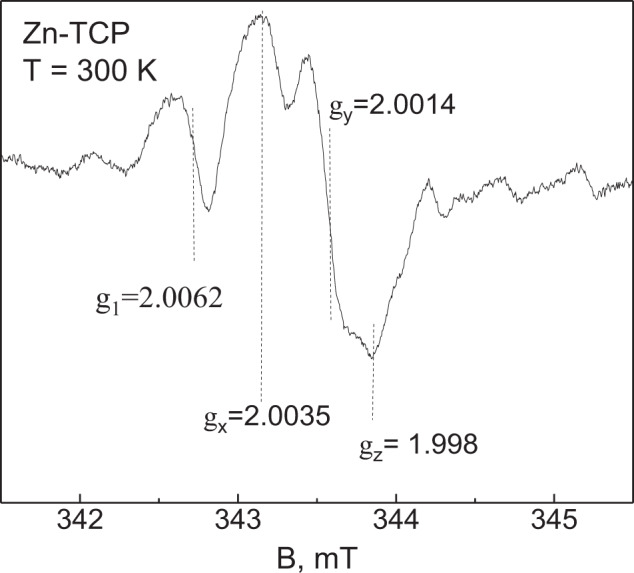
Central part of the X-Ray induced EPR for Zn-TCP powder sample annealed at 900 °C after 512 scans. The calculated values of g-factors are shown

Another group of signals detected around the *g* ≈ 2.00 (Fig. 4) can be ascribed to the presence of two paramagnetic centers [48]. One with the g-factor of *g*_1_ = 2.0062 is probably due to the PO_4_^2−^ stable radical formed by electron ejection from the PO_4_^3−^ group [49]. Usually, one detects PO_4_^2−^ radicals of axial symmetry [50] with *g*_⊥_ = 2.0062 and *g*_||_ = 2.0134–2.030. Probably, *g*_||_ coincides with one of the components of the second signal with the values of *g*_x_ = 2.0035, *g*_y_ = 2.0014, *g*_z_ = 1.998, typical for the carbonate radical CO_2_^−^ of orthorhombic symmetry in various apatite matrices [48, 50], substituting PO_4_^3−^ group [51].

**Fig. 5:**
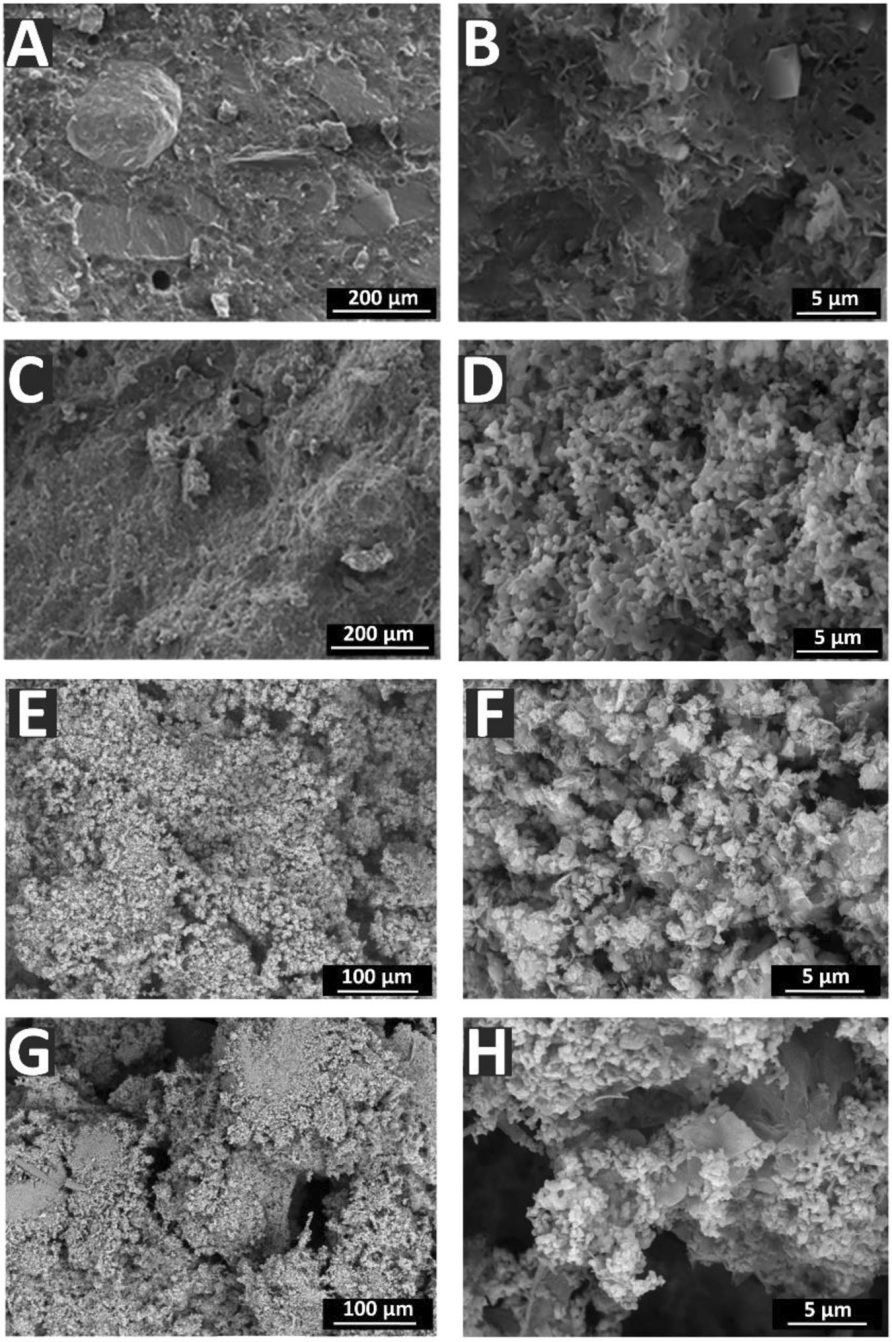
Cements’ morphology: **A**, **B** cement based on β-TCP; **C**, **D** cement based on Zn-β-TCP; **E**, **F** cement based on β-TCP after soaking in physiological solution; **G**, **H** cement based on Zn-β-TCP after soaking in physiological solution

The morphology of cements investigated by SEM is shown in Fig. 5. The cements obtained with non-substituted β-TCP (A, B) are characterized by a large particle morphology compared to the cements obtained with Zn-β-TCP (C, D), having a finer surface aspect. This could be connected to an increase in dispersion when Zn^2+^ ions are introduced.

Soaking in physiological solution during 60 days led to the change in the morphology of samples (see Fig. 5E–H). The samples have a non-uniform microstructure, with some large particles and newly formed smaller particles on them. According to the XRD, after soaking, the detected phases were DCPD and HA (Fig. 1B, D). The FTIR analysis revealed that for the cements based on non-substituted β-TCP, the intensities of peaks assigned to hydroxyl and carbonate groups decreased after keeping in physiological solution, while for cements based on Zn-TCP, the intensities of these peaks increased (see Fig. 2B, C). This experimental evidence could likely be explained by a finer, nanoparticle nature of the Zn-β-TCP cement, compared to non-substituted β-TCP. Smaller particles interact faster with the physiological solution, so the intensity of the peaks of the carbonate and hydroxyl groups increased in the FTIR spectra.

The compressive strength for cement sample cylinders made of non-substituted β-TCP and of Zn-β-TCP was measured 7 days after their setting. According to the experimental results, the average compressive strength for the Zn-β-TCP cements was about 17.5 ± 1.6 MPa, whereas for the non-substituted cements about 15.4 ± 1.6 MPa. Therefore, an increase in the compressive strength for the substituted Zn-β-TCP cement is attested. A similar result was obtained in our previous work [14], in which iron substituted TCP cement was investigated. Also in that case the compressive strength for substituted cement was higher compared to the non-substituted one. Such a difference can probably be explained by a finer and denser structure of the Zn-β-TCP cement compared to the non-substituted β-TCP cement (see SEM observations in Fig. 5A, C). Usually, a higher strength is observed for cements with a denser structure, i.e., with more contacts between particles [28].

The compressive strength of the cements also changed with the time elapsed after mixing of the powder and the hardening liquid. A few minutes after setting, it was 0.5–1 MPa, and 1 h after setting—1–2 MPa. After 1 day, the compressive strength increased to 2–3 MPa, and the maximum strength of about 19 MPa, was reached after 7 days of setting.

**Fig. 6:**
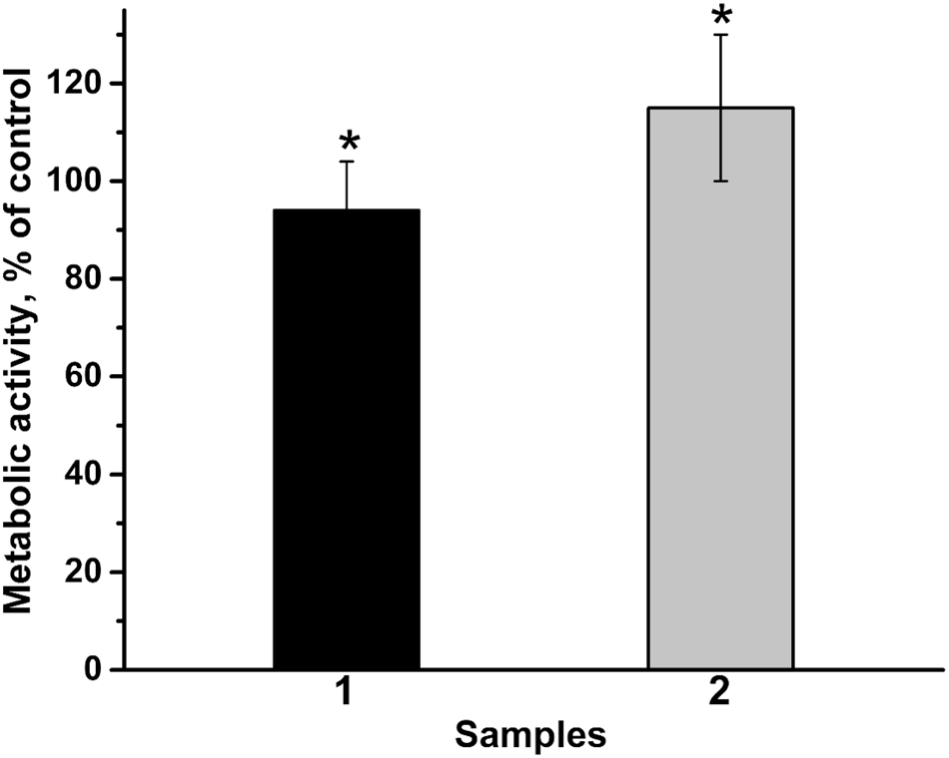
MTT test: NCTC L929 cells’ metabolic activity during 24 h of incubation with extracts from materials: 1-β-TCP, 2-Zn-β-TCP cement (*p < 0.01). Control sample (glass slide) is 100%. The error bars correspond to average ± standard deviation

The investigation of the metabolic activity of the NCTC L929 cells in the developed material extracts was carried out using the MTT test (Fig. 6). Due to the hydrolysis of brushite, which leads to the formation of orthophosphoric acid, the extracts were acidified. To solve this issue, 0.1 M of NaOH was added to adjust pH up to 7.4.

The MTT test showed a significant difference (*p* ≤ 0.01) between the samples of β-TCP and Zn-β-TCP cements. As can be seen from Fig. 6, the introduction of Zn in the cement contributed to the increase in the cell viability in about 10% with respect to the non-substituted cement, which can be explained by the positive effect of Zn^2+^ ions on the proliferation of fibroblast cells. It is likely due to the fact that zinc plays an important role in various biological processes and participates in a number of metabolic functions, necessary for growth and development of cells. Indeed, in bone tissue, Zn^2+^ ions have been proven to promote osteoclast apoptosis, reduce their activity and inhibit their adhesion to the β‐TCP, blocking early and much greater absorption of β‐TCP, providing favorable conditions for new bone formation [52].

**Fig. 7:**
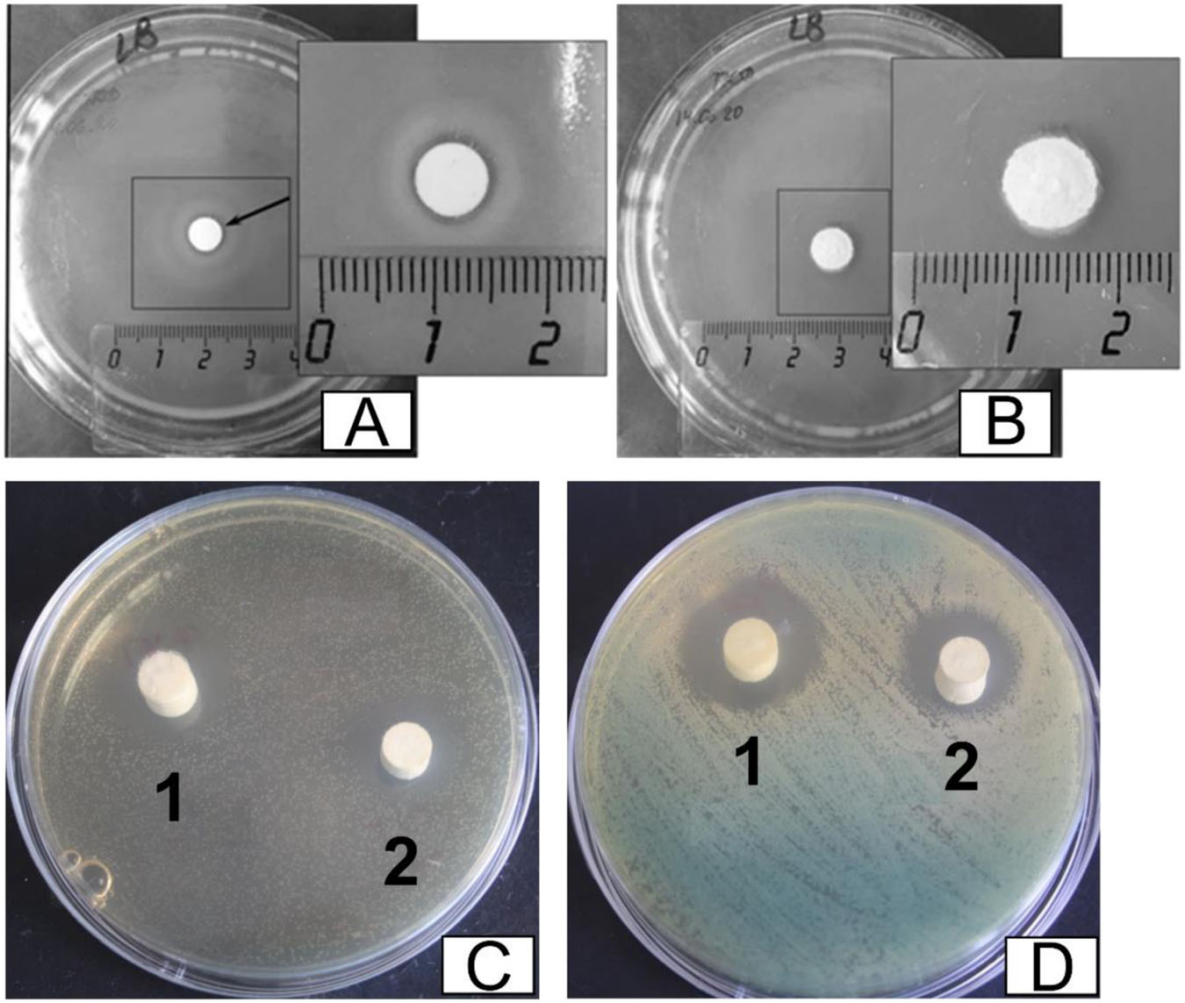
The inhibition of growth of: *E. Coli* for **A** Zn-TCP cement and **B** TCP cement; **C** *E. faecium*, and **D** *P. aeruginosa* for Zn-TCP cement (1) and TCP cement (2)

We also investigated the antibacterial activity of the developed brushite cements, the obtained results are reported in Fig. 7 and Table 2. As can be observed from the obtained experimental data, both the TCP and Zn-TCP cements exhibit antibacterial activity. As it is evidenced in Table 2, the Zn-substituted TCP cement shows a stronger antibacterial activity, which is expressed in a larger bacterial zone of inhibition, with respect to the TCP cement (see Table 2). This conclusion is valid for all the three tested bacteria species: *E. Coli*, *E. faecium*, and *P. aeruginosa*, which are the most frequent in orthopedic surgery [53].

**Table 2:** Antibacterial activity of the cements against *E. coli*, *E. faecium*, and *P. aeruginosa*

Sample	Inhibition zone, mm		
	*E. Coli*	*E. faecium*	*P. aeruginosa*
TCP cement	0	18	18
Zn-TCP cement	14	24	23

Postsurgical infection is a common and serious complication that leads to reduced quality of life, multiple surgeries, and high treatment costs. Indeed, postsurgical infection after osteosynthesis has an incidence of 0.5–10% for closed fracture [54] and up to 50% for open fractures [55]. It occurs in 1–14% of patients after spine surgery [56] and has similar incidence for several orthopedic and trauma procedures involving implantable devices [57]. The socioeconomic burden and management of such infections are incredibly challenging. Therefore, the development of innovative materials with intrinsic antibacterial properties and capable of enhancing osteointegration is desirable for clinical use. By adding Zn^2+^ ion, we aim to stimulate bone metabolism, cell adhesion [58] inhibit bacterial growth to avoid infections and enhance bone formation, regeneration, and mineralization [59].

In [60], we demonstrated that silicon-substituted TCP possesses antibacterial activity against *E. Coli*. In that work, it is reported that when the diameter of inhibition zone is about 10–15 mm, the material is characterized by a weak antibacterial activity, about 15–20 mm—by a moderate one, and if more than 20 mm—by a significant antibacterial activity. Based on this, it is possible to conclude that the TCP cement is not active against *E. Coli* and moderately active against *E. faecium* and *P. aeruginosa*, whereas Zn-TCP cement possesses weak antibacterial activity against *E. Coli* and is significantly active against *E. faecium* and *P. aeruginosa*.

Based on our results it can be concluded that the Zn-β-TCP cement has positive effect on the cell viability and, at the same time, has a negative effect on bacteria. These properties make this material an ideal candidate for use in the fabrication of orthopedic implants.

## Conclusions

In this work, the cement based on the Zn-substituted β-TCP powder with a simplified preparation recipe and improved characteristics was developed. Its properties were studied in comparison with the reference non-substituted β-TCP cement. The initial cement powder was composed of equimolecular mixture of β-TCP and MCPM, and the hardening liquid—of 8% of aqueous solution of citric acid containing 30% of ammonium citrate. The final components of the cements were DCPD and β-TCP. The setting time of cements was 8 min (the ratio of cement powder: hardening liquid = 3:1), which is optimal for preparation and application of the developed cements for bone defects during surgery. The Zn^2+^ content was selected to be 1.40 wt%. The pH of the cements reached 6.5 within 60 min after setting. After soaking in physiological solution for 60 days, the morphology and composition of cements changed. The final phases were DCPD and HA.

The EPR measurements showed the presence of the trapped hydrogen and confirmed that annealing at 900 °C led to the significant reduction of carbonate impurities embedded into the β-TCP structure.

The average compressive strength for the Zn-β-TCP cement was about 17.5 ± 1.6 MPa, whereas for the non-substituted β-TCP cement—about 15.4 ± 1.6 MPa (measured 7 days after cements’ setting). Therefore, the Zn-addition led to the enhancement of the mechanical properties of the β-TCP cement.

The NCTC L929 fibroblast cell viability on the developed Zn-β-TCP cement was 10% higher compared to cement without Zn and possess antibacterial properties against *E. coli, E. faecium*, and *P. aeruginosa*.

β-TCP is widely used in the clinics. It can be readily doped with ions, which may provide functionalities like bactericidal activity. Indeed, the developed Zn-substituted β-TCP cement is promising in the clinical setting in view of its significant antibacterial activity, improved mechanical properties and enhanced cell viability. This finding confirms that the novel material could be a valid strategy for a range of biomedical application in humans. Therefore, it could offer promising potential for bone replacement and repair in moderate and non-load-bearing defects that are prone to infection in orthopedic and trauma setting. Despite these promising properties, the amount of the substitution ion should be carefully optimized, in order to enhance the antibacterial activity, trying to maintain the positive effect on cells.

## References

[1] Palangkaraya A, Yong J (2009). Population ageing and its implications on aggregate health care demand: empirical evidence from 22 OECD countries. Int J Health Care Financ Econ.

[2] Langer R, Tirrell DA (2004). Designing materials for biology and medicine. Nature.

[3] Betz RR (2002). Limitations of autograft and allograft: new synthetic solutions. Orthopedics..

[4] Seiler JG, Johnson J (2000). Iliac crest autogenous bone grafting: donor site complications. J South Orthop Assoc.

[5] Drosse I, Volkmer E, Capanna R, Biase PD, Mutschler W, Schieker M (2008). Tissue engineering for bone defect healing: an update on a multi-component approach. Injury.

[6] Holzapfel BM, Reichert JC, Schantz J-T, Gbureck U, Rackwitz L, Nöth U (2013). How smart do biomaterials need to be? A translational science and clinical point of view. Adv Drug Del Rev.

[7] Hanson B, van der Werken C, Stengel D (2008). Surgeons’ beliefs and perceptions about removal of orthopaedic implants. BMC Musculoskel Disord.

[8] Bohner M, Santoni BLG, Döbelin N (2020). β-tricalcium phosphate for bone substitution: synthesis and properties. Acta Biomater.

[9] Antoniac IV, Filipescu M, Barbaro K, Bonciu A, Birjega R, Cotrut CM (2020). Iron ion‐doped tricalcium phosphate coatings improve the properties of biodegradable magnesium alloys for biomedical implant application. Adv Mater Interfaces.

[10] Rau JV, Fosca M, Fadeeva IV, Kalay S, Culha M, Raucci MG (2020). Tricalcium phosphate cement supplemented with boron nitride nanotubes with enhanced biological properties. Mater Sci Eng C.

[11] Seidenstuecker M, Kerr L, Bernstein A, Mayr H, Suedkamp N, Gadow R (2018). 3D powder printed bioglass and β-Tricalcium phosphate bone scaffolds. Materials.

[12] Viana ÍEL, Lopes RM, Silva FRO, Lima NB, Aranha ACC, Feitosa S (2020). Novel fluoride and stannous -functionalized β-tricalcium phosphate nanoparticles for the management of dental erosion. J Dent.

[13] Kopylov P, Jonsson K, Thorngren KG, Aspenberg P (2016). Injectable calcium phosphate in the treatment of distal radial fractures. J Hand Surg Am.

[14] Uskoković V, Graziani V, Wu VM, Fadeeva IV, Fomin AS, Presniakov IA (2019). Gold is for the mistress, silver for the maid: Enhanced mechanical properties, osteoinduction and antibacterial activity due to iron doping of tricalcium phosphate bone cements. Mater Sci Eng, C.

[15] Wu T, Shi H, Liang Y, Lu T, Lin Z, Ye J (2020). Improving osteogenesis of calcium phosphate bone cement by incorporating with manganese doped β-tricalcium phosphate. Mater Sci Eng, C.

[16] Graziani V, Fosca M, Egorov AA, Zobkov YV, Fedotov AY, Baranchikov AE (2016). Zinc-releasing calcium phosphate cements for bone substitute materials. Ceram Int.

[17] Gallinetti S, Canal C, Ginebra MP, Ferreira J (2014). Development and characterization of biphasic hydroxyapatite/β‐TCP cements. J Am Ceram Soc.

[18] Son Y-J, Lee I-C, Jo H-H, Chung T-J, Oh K-S (2019). Setting behavior and drug release from brushite bone cement prepared with granulated hydroxyapatite and β-tricalcium phosphate. J Korean Ceram Soc.

[19] Honda M, Kawanobe Y, Nagata K, Ishii K, Matsumoto M, Aizawa M (2020). Bactericidal and bioresorbable calcium phosphate cements fabricated by silver-containing tricalcium phosphate microspheres. Int J Mol Sci.

[20] Bettger WJ, O’Dell BL (1993). Physiological roles of zinc in the plasma membrane of mammalian cells. J Nutr Biochem.

[21] Ovesen J, Møller-Madsen B, Thomsen JS, Danscher G, Mosekilde L (2001). The positive effects of zinc on skeletal strength in growing rats. Bone..

[22] Gür A, Çolpan L, Nas K, Çevik R, Saraç J, Erdoğan F (2002). The role of trace minerals in the pathogenesis of postmenopausal osteoporosis and a new effect of calcitonin. J Bone Min Metab.

[23] Wang T, Zhang J-C, Chen Y, Xiao P-G, Yang M-S (2007). Effect of zinc ion on the osteogenic and adipogenic differentiation of mouse primary bone marrow stromal cells and the adipocytic trans- differentiation of mouse primary osteoblasts. J Trace Elem Med Biol.

[24] Kimura E, Kikuta E (2000). Why zinc in zinc enzymes? From biological roles to DNA base-selective recognition. J Biol Inorg Chem.

[25] Uskoković V, Wu V (2016). Calcium phosphate as a key material for socially responsible tissue engineering. Materials.

[26] Tannoury CA, An HS (2014). Complications with the use of bone morphogenetic protein 2 (BMP-2) in spine surgery. Spine J.

[27] Hustedt JW, Blizzard DJ (2014). The controversy surrounding bone morphogenetic proteins in the spine: a review of current research. Yale J Biol Med.

[28] Fadeeva IV, Gafurov MR, Kiiaeva IA, Orlinskii SB, Kuznetsova LM, Filippov YY (2016). Tricalcium phosphate ceramics doped with silver, copper, zinc, and iron (III) ions in concentrations of less than 0.5 wt.% for bone tissue regeneration. BioNanoScience.

[29] Zhang L, Guo J, Yan T, Han Y (2018). Fibroblast responses and antibacterial activity of Cu and Zn co-doped TiO2 for percutaneous implants. Appl Surf Sci.

[30] Bakhsheshi-Rad HR, Hamzah E, Low HT, Kasiri-Asgarani M, Farahany S, Akbari E (2017). Fabrication of biodegradable Zn-Al-Mg alloy: mechanical properties, corrosion behavior, cytotoxicity and antibacterial activities. Mater Sci Eng C.

[31] Walczyk D, Malina D, KrÓL M, Pluta K, Sobczak-Kupiec A (2016). Physicochemical characterization of zinc-substituted calcium phosphates. Bull Mater Sci.

[32] Boanini E, Gazzano M, Nervi C, Chierotti MR, Rubini K, Gobetto R (2019). Strontium and zinc substitution in β-tricalcium phosphate: an X-ray diffraction, solid state NMR and ATR-FTIR study. J Funct Biomater.

[33] Pina S, Ferreira JMF (2010). Brushite-forming Mg-, Zn- and Sr-substituted bone cements for clinical applications. Materials.

[34] Ito A, Ojima K, Naito H, Ichinose N, Tateishi T (2000). Preparation, solubility, and cytocompatibility of zinc-releasing calcium phosphate ceramics. J Biomed Mater Res.

[35] Sogo Y, Ito A, Kamo M, Sakurai T, Onuma K, Ichinose N (2004). Hydrolysis and cytocompatibility of zinc-containing α-tricalcium phosphate powder. Mater Sci Eng C.

[36] Li X, Sogo Y, Ito A, Mutsuzaki H, Ochiai N, Kobayashi T (2009). The optimum zinc content in set calcium phosphate cement for promoting bone formation in vivo. Mater Sci Eng C.

[37] Fadeeva IV, Teterina A, Komlev V, Barinov SM (2012). Biodegradable bone cement based on β- tricalcium phosphate. Mater Sci Appl Res.

[38] GOST R ISO 10993-11-2011 Medical devices. Biological evaluation of medical devices. Part 11. Tests for systemic toxicity.

[39] GOST ISO 10993-12-2015 Medical devices. Biological evaluation of medical devices. Part 12. Sample preparation and reference materials.

[40] Barralet J, Best S, Bonfield W (1998). Carbonate substitution in precipitated hydroxyapatite: an investigation into the effects of reaction temperature and bicarbonate ion concentration. J Biomed Mater Res.

[41] Barinov SM, Rau JV, Fadeeva IV, Cesaro SN, Ferro D, Trionfetti G (2006). Carbonate loss from two magnesium-substituted carbonated apatites prepared by different synthesis techniques. Mater Res Bull.

[42] Rau JV, Cesaro SN, Ferro D, Barinov SM, Fadeeva IV (2004). FTIR study of carbonate loss from carbonated apatites in the wide temperature range. J Biomed Mater Res.

[43] Novikov RG, Konopnitskii R, Tsyganenko AA (2018). Distortions in IR spectra related to registration conditions: II. The influence of scattering. Opt Spectrosc.

[44] Gabbasov B, Gafurov M, Starshova A, Shurtakova D, Murzakhanov F, Mamin G (2019). Conventional, pulsed and high-field electron paramagnetic resonance for studying metal impurities in calcium phosphates of biogenic and synthetic origins. J Magn Magn Mater.

[45] Cavalu S, Popa A, Bratu I, Borodi G, Maghiar A (2015). New evidences of key factors involved in “Silent Stones” etiopathogenesis and trace elements: microscopic, spectroscopic, and biochemical approach. Biol Trace Elem Res.

[46] Matković I, Maltar-Strmečki N, Babić-Ivančić V, Dutour Sikirić M, Noethig-Laslo V (2012). Characterisation of β-tricalcium phosphate-based bone substitute materials by electron paramagnetic resonance spectroscopy. Radiat Phys Chem.

[47] Nakashima K, Yamauchi J (2005). ESR Investigation of a stable trapped hydrogen atom in X-ray-irradiated β-tricalcium phosphate at room temperature. J Am Chem Soc.

[48] Murray KA, Collins MN, O’Sullivan RP, Ren G, Devine DM, Murphy A (2018). Influence of gamma and electron beam sterilization on the stability of a premixed injectable calcium phosphate cement for trauma indications. J Mech Behav Biomed Mater.

[49] Sadlo J, Matthys P, Vanhaelewyn G, Callens F, Michalik J, Stachowicz W (1998). EPR and ENDOR of radiation-induced CO33- radicals in human tooth enamel heated at 400°C. J Chem Soc Faraday Trans.

[50] Fisher BV, Morgan RE, Phillips GO, Wardale HW (1971). Radiation damage in calcium phosphates and collagen: an interpretation of ESR spectra. Radiat Res.

[51] Schramm DU, Rossi AM (2000). Electron spin resonance (ESR) studies of radicals in irradiated A and B-type carbonate-containing apatites. Appl Radiat Isot.

[52] Yamada Y, Ito A, Kojima H, Sakane M, Miyakawa S, Uemura T (2007). Inhibitory effect of Zn2+ in zinc‐containing β‐tricalcium phosphate on resorbing activity of mature osteoclasts. J Biomed Mater Res Part A.

[53] Cavalu S, Simon V, Goller G, Akin I (2011). Bioactivity and antimicrobial properties of PMMA/Ag 2O acrylic bone cement collagen coated. Dig J Nanomater Bios.

[54] Bonnevialle P, Bonnomet F, Philippe R, Loubignac F, Rubens-Duval B, Talbi A (2012). Early surgical site infection in adult appendicular skeleton trauma surgery: a multicenter prospective series. Orthop Traumatol Surg Res.

[55] Oliveira PR, Carvalho VC, da Silva Felix C, de Paula AP, Santos-Silva J, Lima ALLM (2016). The incidence and microbiological profile of surgical site infections following internal fixation of closed and open fractures. Rev Brasileira de Ortop (Engl Ed).

[56] Shillingford JN, Laratta JL, Reddy H, Ha A, Lehman RA, Lenke LG (2018). Postoperative surgical site infection after spine surgery: an update from the Scoliosis Research Society (SRS) morbidity and mortality database*. Spine Deform.

[57] Gupta R, Sood M, Malhotra A, Masih G, Raghav M, Khanna T (2018). Incidence, risk factors, and management of infection following anterior cruciate ligament reconstruction surgery. Indian J Orthop.

[58] Webster TJ, Ergun C, Doremus RH, Bizios R (2002). Hydroxylapatite with substituted magnesium, zinc, cadmium, and yttrium. II. Mechanisms of osteoblast adhesion. J Biomed Mater Res.

[59] Luo X, Barbieri D, Davison N, Yan Y, de Bruijn JD, Yuan H (2014). Zinc in calcium phosphate mediates bone induction: in vitro and in vivo model. Acta Biomater.

[60] Fadeeva IV, Filippov YY, Fomin AS, Shaposhnikov ME, Davydova GA, Antonova OS (2015). Synthesis of micro- and nanosized bioresorbing silicon-substituted tricalcium phosphates for bone tissue engineering and their biological safety using mesenchymal stem cells. Nanomech Sci Technol: Int J.

